# Technical, economic, and environmental feasibility of rice hull ash from electricity generation as a mineral additive to concrete

**DOI:** 10.1038/s41598-024-59615-1

**Published:** 2024-04-22

**Authors:** Jin Wook Ro, Patrick R. Cunningham, Sabbie A. Miller, Alissa Kendall, John Harvey

**Affiliations:** 1 Institute of Transportation Studies, University of California, Davis, USA; 2 Department of Civil and Environmental Engineering, University of California, Davis, USA

**Keywords:** Environmental impact, Climate-change mitigation, Sustainability

## Abstract

A circular economy based on symbiotic relationships among sectors, where the waste from one is resource to another, holds promise for cost-effective and sustainable production. This research explores such a model for the agriculture, energy, and construction sectors in California. Here, we develop new an understanding for the synergistic utilization mechanisms for rice hull, a byproduct from rice production, as a feedstock for electricity generation and rice hull ash (RHA) used as a supplementary cementitious material in concrete. A suite of methods including experimental analysis, techno-economic analysis (TEA), and life-cycle assessment (LCA) were applied to estimate the cost and environmental performance of the system. TEA results showed that the electricity price required for break even on expenses without selling RHA is $0.07/kWh, lower than the market price. As such, RHA may be available at little to no cost to concrete producers. Our experimental results showed the viability of RHA to be used as a supplementary cementitious material, meaning it can replace a portion of the cement used in concrete. LCA results showed that replacing 15% of cement with RHA in concrete can reduce carbon dioxide equivalent (CO_2_e) emissions by 15% while still meeting material performance targets. While the substitution rate of RHA for cement may be modest, RHA generated from California alone could mitigate 0.2% of total CO_2_e from the entire cement production sector in the United States and 1% in California.

## Introduction

Concrete is the most widely used material for infrastructure and buildings, consumed at a rate of 1.4 cubic meters per person worldwide annually^1^. The production of cement, the binding material in concrete, accounts for 5–8% of global carbon dioxide (CO_2_) emissions, emitting between 0.66 and 0.82 kg of CO_2_ per kilogram of cement production^1,2^. In the United States, the cement production sector alone was responsible for 40.7 million metric tons (MMT) of CO_2_ in 2020, about 0.8% of total greenhouse gas (GHG) emissions and 11% of GHG emissions from the industrial sector^3^. The primary source of these emissions is from producing clinker, a kilned and quenched precursor to cement, which is inter-ground with other minerals to form cement. Due to the high carbon intensity of cement and the attendant impacts embodied in concrete, there have been efforts to decrease the GHG emissions and other environmental impacts from cement, concrete, and the construction industry more broadly^4,5^. The use of supplementary cementitious materials (SCMs), such as coal fly ash or slag from iron production, to displace some clinker content in concrete is a widely used solution to decrease the environmental impacts and improve performance characteristics of concrete^6–9^. However, the availability of materials like fly ash are becoming highly constrained in certain areas because of closures of coal power plants^10^.

In this work, we focus on energy, agriculture, and cement demand in California. California is a well-suited case study due to recent legislation mandating the decarbonization of energy and building materials production^11–13^. In 2020, California’s cement production emitted 7.5 MMT CO_2_, comprising about 2% of total and 10% of the industrial sector GHG emissions^14^. As the second largest cement producing state in the United States, significant amounts of SCMs are consumed within California, recently estimated at about 1 MMT of fly ash are consumed as SCM annually^15^, but all fly ash is imported since there are no coal-fired power plants in California. The demand for SCMs in California is expected to increase along with the increase of the demand for cement in California by 65% by 2050^16,17^.

Meanwhile, the combustion of solid biomass has been widely used for renewable, low-carbon heat and electricity generation. In California, about 3% of electricity generated in-state is from biomass^18,19^. Similar to coal burning, the combustion of solid biomass generates a large amount of ash from the inorganic components of the biomass and has a potential to be used as a pozzolanic material^20–22^. Among the available biomass resources, this study examines rice hulls as a potential candidate for biomass-derived energy generation and for formation of rice hull ash (RHA) to replace the conventional SCMs by establishing the viability of RHA as an SCM in concrete mixtures via experimental analysis, and then examining the cost and environmental performance of RHA used as an SCM from a life cycle perspective.

Rice hulls present a unique opportunity. California is the second largest rice-growing state in the United States. It has a long history of utilizing rice hulls, a byproduct of rice milling, to generate renewable electricity, and the resulting RHA has potential to be used as an SCM. Further, the research community has previously investigated the potential of RHA as an SCM and its environmental and economic feasibility^23–25^. Hu et al.^26^ showed that mixing RHA with cement reduced embodied CO_2_ emission and energy consumption while sustaining the required compressive strength and sulfate resistance, and the comprehensive data analysis by Ozturk et al.^27^ concluded that it is possible to decrease CO_2_ emissions by 25% and increase the cost efficiency of concrete by 65% by using RHA and other pozzolanic materials at optimal conditions. Fernando et al.^28^ also showed that the utilization of RHA could reduce environmental impacts providing significant benefits in terms of fresh and marine water ecotoxicity along with reducing total GHG emissions. Additionally, as rice is one of the most common agricultural products worldwide, sufficient understanding of potential symbiotic pathways to meet needs for food, energy, and materials is critical to advancing our ability to develop a circular economy. However, there is yet insufficient information on the costs of the processes that generate RHA from combustion for electricity generation and the technical and environmental performance of RHA as an SCM in California.

To determine the feasibility and economic and environmental performance of valorizing rice hulls in this way, the following research questions must be answered:

1. What are the current costs and operating conditions for rice hull derived electricity in California?
2. What are the expected costs and availability of RHA from electricity generation in California?
3. How do concrete mixes that contain RHA perform?
4. What are the environmental impacts of producing RHA and how do they compare to the current environmental impacts of more common SCMs such as coal fly ash?

To answer these research questions, a suite of research methods was required including techno-economic analysis (TEA), experimental testing methods, and life cycle assessment (LCA). First, the economic feasibility and availability of electricity generation from rice hulls and RHA production were analyzed using TEA. The cost of electricity from a rice hull power plant was calculated based on assumptions from the literature and an existing facility, and the economic feasibility with different RHA prices and rice hull costs were analyzed as well. After assessing the economic feasibility and availability, the performance of concrete mixtures with RHA was measured using compressive testing, flexural testing and rapid chloride permeability testing, and the results were compared to a regular concrete mixture with 100% Portland cement (PC). Then, the environmental impacts of concrete mixtures with RHA were quantified using LCA. The environmental impacts calculated from LCA were allocated among the co-products generated by the power plant, which are electricity and RHA, and the results for RHA were compared to PC and fly ashes which RHA can replace. Detailed processes and assumptions are described in the Methods section.

## Results and discussion

### Economic feasibility results

Here we present a cost estimate for generating electricity from rice hulls, summarized in Table 1. If all rice hulls produced in California, estimated at 393,945 metric tons (tonne) of rice hulls in 2018, were used to generate electricity, approximately 390 GWh of electricity would be generated and 79,800 tonne of RHA would have been generated in that year, assuming 1.01 tonne of rice hulls are required to produce 1 MWh of electricity. The electricity price required for break-even operation was found to be $0.07/kWh without revenue from selling RHA, significantly lower than the retail value of electricity in California at $0.15/kWh^29^. Thus, electricity generation from rice hull is economically feasible even without selling the RHA which is generated as a byproduct.

**Table 1:** Electricity generation and cost analysis results.

Parameter		Unit
Total feedstock available in CA	393,945	tonne/year
Total electricity generation	390,006	MWh/year
Total ash production	79,800	tonne/year
Capital expenses (CAPEX)	119,426,000	$
Operating expenses (OPEX)	4,009,000	$/year
Electricity price	0.070	$/kWh
Required ash price	0	$/tonne

Assuming all available feedstocks generated in California were converted to electricity and ash ($, 2019).

These findings suggest that a power plant using rice hulls exceeds break-even costs without any value from RHA, which indicates that RHA could be available to the concrete industry at little to no cost beyond transportation. This limited expense is a notable benefit as currently the market drivers in California have supported substantial prices for many SCMs. However, there are several uncertainty parameters that should be considered, including that the cost of power plant operation is the cost of rice hull acquisition. When the price of electricity is assumed at $0.15/kWh, the maximum viable cost to the power plant for rice hull is $81/tonne. In California, the price of rice hull has historically fluctuated between $3.31/tonne and $5.51/tonne since 2017, but recently has increased to $11.02^30^. Even at its highest price, it is still lower than the maximum viable cost of $81/tonne. Alternatively, RHA could be a value to the power plant, considering that other SCMs such as fly ash retail at around $83/tonne^31^. At this price for RHA, the power plant can remain profitable with rice hull costs below $98/tonne and break-even at rice hull costs of $98/tonne.

### Experimental analysis results

**Figure 1:**
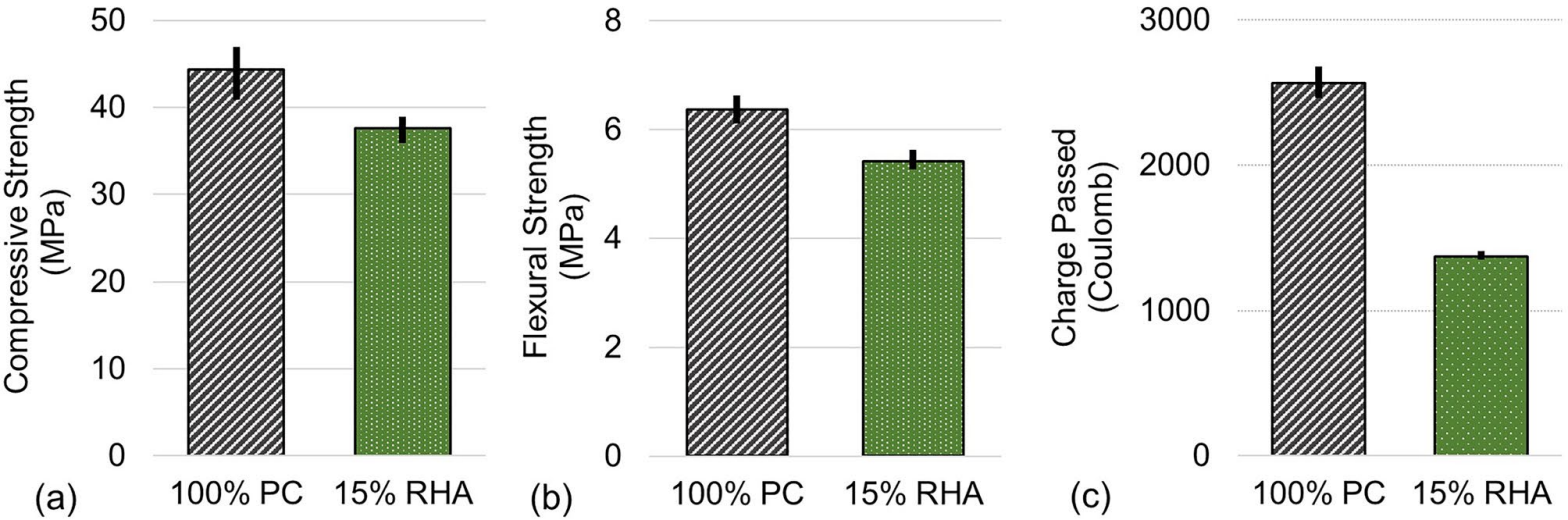
Material performance and durability indicators of concrete with only PC binder (100% PC) and concrete with 15% PC replaced with RHA (15% RHA). (**a**) Average compressive strength (n = 5), (**b**) average flexural strength (n = 3), and (**c**) average charge passed during rapid chloride ingress (n = 3). Lower charge passed indicates higher resistance to chloride ion penetration, which may improve durability. In all plots, bars indicate range of measurements.

A 15% replacement of PC with RHA acquired from conventional bioenergy production resulted in moderate changes to key material performance characteristics. Reductions were noted for 28-day compressive and flexural strength (Fig. 1a,b), leading to approximately 15% lower strength in both cases than if PC alone was used as the cementitious binding powder. While a reduction in strength is not necessarily desirable, this 15% reduction is less than the reduction in strength that would occur from the removal of 15% of cement with no replacement. Further, we note if other mixture proportions were varied, a 15% RHA mixture can exceed the strength of a 15% fly ash replacement mixture^32^. The likely reason for these shifts in properties is that the RHA could be acting as a pozzolan that contributes to later-age strength, but less so to early strength. Other works have demonstrated that, at 56 days, strength loss can be much less (~ 1–5%) for rice hulls^25^. With grinding, RHA has been shown to become more reactive^33^. If early age strength was desirable, post-combustion processing could be used to enhance desirable properties. Notably, the use of RHA in concrete led to a significant benefit of reduced chloride ion permeability (Fig. 1c), indicating greater resistance to chloride ingress. Chloride ingress is a durability concern, particularly in coastal areas, which are where ~ 40% of human populations live^34,35^. The RHA specimens passed approximately half the charge of the control specimens, showing an improvement in the Chloride Ion Penetrability Category defined by ASTM C1202^36^. This result agrees with those reported in literature^37^. Thus, even with this less reactive RHA, the permeability of concrete to chloride ingress could be substantially reduced from the “Moderate” permeability range (2000–4000 coulombs) exhibited by the control concrete to the “Low” permeability range (1000–2000 coulombs) exhibited by the RHA concrete. RHA may lead to concrete that is more durable when exposed to higher chloride ion conditions than concrete with only PC, which can reduce the negative consequences of steel corrosion and can increase the longevity of structures.

### Environmental impacts results

In this study, electricity and RHA are the products from the rice hull power plant, and environmental impacts are considered for both products. Because multiple products are formed, here we consider allocation, referring to the partitioning of the impacts among co-products generated from the system. Since physical relationships between electricity and RHA cannot be established due to their different characteristics and functionalities, the economic allocation method, which partitions impacts based on their economic value, was used in this study as it is widely used in LCA^38^. Since the price of RHA from this study is $0/tonne, all environmental impacts are allocated to electricity. However, the price of $0 calculated in this study does not imply that the value of RHA is zero. Considering that RHA is replacing other SCMs, it is possible to assume that RHA has the same economic value as other SCMs such as fly ash from coal electricity or ground blast furnace slag from the iron industry. Thus, $83/tonne, an estimate of the value of fly ash, is used as the economic value of the RHA^31^. In Table 2, the total economic value of electricity and RHA are shown assuming utilization of all rice hulls in California for power generation and ash production, and the assigned environmental impacts based on the economic values are shown in Table 3.

**Table 2:** Economic value estimates for electricity and RHA.

	Electricity	RHA
Generation/production	390,006 MWh/year	79,814 tonne/year
Unit price	$0.070/kWh	$83/tonne
Total economic value	$27,300,420/year	$6,624,562/year
Total economic value	80%	20%

**Table 3:** Environmental impacts of electricity and RHA per unit output (*DCB* dichlorobenzene, *CFC* chlorofluorocarbon).

Impact category	Electricity	RHA	Unit
Unit output	1 MWh	1 tonne	
Abiotic depletion, elements	4.30E−05	1.08E−05	kg antimony (Sb) eq.
Abiotic depletion fossil	380	95	MJ
Acidification	0.888	0.222	kg SO_2_ eq.
Eutrophication	0.226	0.566	kg PO_4_ eq.
Freshwater aquatic ecotoxicity	1.13	0.28	kg DCB eq.
Global warming	33.2	8.3	kg CO_2_ eq.
Human toxicity	6.53	1.63	kg DCB eq.
Marine aquatic ecotoxicity	32,200	8100	kg DCB eq.
Ozone depletion	1.07E−13	2.68E−14	kg CFC-11 eq.
Photochemical ozone creation	0.163	0.041	kg C_2_H_4_ eq.
Terrestrial ecotoxicity	1.09	0.27	kg DCB eq.

In Table 4, environmental impacts of PC, PC with 5–15% fly ash, PC with 15–40% fly ash, RHA, and PC with 15% RHA are shown. The results show that the RHA has lower environmental impacts in all impact categories than other cement products, and the mixture also has lower environmental impacts than PC or PC with 5–15% fly ash. For example, the mixture of 85% PC and 15% RHA, which was tested in this study, may lead to 15% reductions in the global warming potential (GWP) and 15% reductions in abiotic fossil depletion compared to PC. If 79,814 tonne of RHA is used as an SCM in lieu of PC annually, 70,771 tonne of CO_2_e could be mitigated per year, which is about 0.2% and 1% of CO_2_e from the cement production sector in the United States and in California, respectively.

**Table 4:** Environmental impacts of Portland cement (PC), PC with 5–15% fly ash, PC with 15–40% fly ash, rice hull ash (RHA), and mixture of 85% PC and 15% RHA (*ADP* Abiotic depletion, *AP* Acidification potential, *EP* eutrophication potential, *FAETP* freshwater aquatic ecotoxicity potential, *GWP* global warming potential, *HTP* human toxicity potential, *MAETP* marine aquatic ecotoxicity potential, *ODP* Ozone layer depletion potential, *POCP* photochemical ozone creation potential, *TETP* terrestrial ecotoxicity potential).

Impact category	Portland cement (PC)	PC, fly ash 5–15%	PC, fly ash 15–40%	RHA	PC, RHA 15%	Unit
Unit output	1	1	1	1	1	tonne
ADP elements	2.03E−03	1.93E−03	1.59E−03	1.08E−05	1.73E−03	kg Sb eq.
ADP fossil	4020	3810	3120	95	3430	MJ
AP	1.59	1.51	1.23	0.222	1.38	kg SO_2_ eq.
EP	0.669	0.637	0.528	0.566	0.577	kg PO_4_ eq.
FAETP	88.9	84.9	71.3	0.28	75.6	kg DCB eq.
GWP	895	848	687	8.3	762	kg CO_2_ eq.
HTP	127	121	103	1.63	108	kg DCB eq.
MAETP	2.55E+05	2.44E+05	2.05E+05	8100	2.18E+05	kg DCB eq.
ODP	2.51E−05	2.38E−05	1.94E−05	2.68E−14	2.13E−05	kg CFC-11 eq.
POCP	0.115	0.109	0.089	0.041	0.104	kg C_2_H_4_ eq.
TETP	1.79	1.70	1.40	0.27	1.56	kg DCB eq.

## Conclusions

This study examined the performance of rice hull, an agricultural residue, for electricity generation with utilization of the resulting combustion ash as an SCM in concrete mix designs from the perspectives of cost and life cycle environmental performance. After verifying the suitability of RHA as an SCM via experimental testing, a cost analysis was conducted and showed the economic feasibility of rice hull as a feedstock for electricity generation and as a source of low-carbon SCM for concrete. Given zero or near-zero cost for rice hulls, electricity generation could be profitable even without revenue from RHA. As such, RHA may be available at no or very low cost to the cement and concrete sector. If the cost of rice hull increases to over $81/tonne, electricity generation may be infeasible without additional revenue from RHA. Assuming the value of RHA at its market price of $83/tonne, the price of coal fly ash (a competing SCM), the cost of rice hull can increase to as much as $98/tonne. LCA results show that RHA has lower environmental impacts than pure PC and PC blended with fly ash, and the impacts reduce on a nearly 1:1 basis with RHA replacement of PC, given the very low environmental impact of RHA.

In summary, for regions where rice hull is a potential fuel for bioenergy, it may also be a potential resource to meet growing demand for SCM for concrete with both economic and environmental feasibility. In addition to this study, further investigation should be considered in a few areas that could improve the feasibility of using RHA in concrete. These include, but are not limited to, geospatial analysis of rice paddy fields, rice millers, and power plants for improving the accuracy of the assessment and analysis on expected consequential impacts on relevant markets. Further investigation into development of pre- and post-treatments to improve RHA qualities and improvement of durability properties of the concrete mixture with RHA should also be considered. Additional work investigating the mechanical properties of RHA materials at ages greater than 28 days and at different scales would be beneficial to understanding the strength development of RHA-cement mixtures and could advance understanding of changes to the micro-mechanical behavior of RHA-cement materials.

## Methods

### Economic feasibility assessment methods

**Figure 2:**
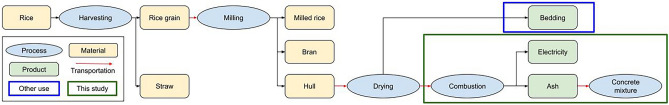
Life cycle of rice hull ash generation.

In this study, the cost and environmental performance of RHA used as an SCM were examined based on the life cycle perspective. The life cycle of RHA used in concrete and the system boundary of this study are shown in Fig. 2.

Rice hulls are the hard husks that cover rice kernels, and as such their availability is a function of annual rice production, which is driven by demand for food resources. Whole rice is harvested and transported to rice milling facilities where rice hulls are separated from the rice kernel. Rice hulls comprise approximately 20% of whole harvested rice by mass^39^. Annual estimates for rice hull generation in California are shown in Table 5 based on the assumptions above.

**Table 5:** Rice hull availability in California^40^*.*

	Unit	2014	2015	2016	2017	2018
Whole rice	tonne	1,720,749	1,718,072	2,149,756	1,690,857	1,969,725
Harvested area	1000 acres	442.14	426.06	536.13	443.25	503.77
Rice yield	metric ton/acre	3.89	4.03	4.01	3.81	3.91
Rice hull (est.)	metric ton	344,150	343,614	429,951	338,171	393,945
Rice hull yield	metric ton/acre	0.78	0.81	0.80	0.76	0.78

Currently, about half of the rice hulls generated in California are used at the Wadham facility in Williams, California, to generate electricity^41^. The Wadham facility has 29.1 MW of maximum plant capacity. It generates about 200,000 MWh annually by combusting rice hulls at 857 °C, selling the electricity to Pacific Gas and Electric^18,42^. For the economic assessment of energy generation, the Wadham facility was used as a reference case.

In this study, there are two potential revenues streams from the power plant: net-generated electricity and RHA. To analyze the economic feasibility of the process, the price of electricity was calculated and then compared to the average retail price of electricity in California. By comparing this, the required revenue from RHA was determined. The price calculation method is described in Eq. (1). The average retail price of electricity was assumed at $0.15/kWh in California^29^.

1 $$Pric{e}_{target}=\frac{Required\, revenu{e}_{levelized\, annual}}{Total\, production},$$

$$Required \,revenu{e}_{levelized\, annual}=\left({\sum }_{year=1}^{Lifetime}\frac{{\left(Required\, revenue\right)}_{year}}{{\left(1+Cost \,of \,money\right)}^{year}}\right)\times Capital \,recovery \,factor,$$

$$Capital\, recovery\, factor=Cost\, of\, mone{y}_{i}\times \frac{{\left(1+Cost \,of \,mone{y}_{i}\right)}^{lifetime}}{({\left(1+Cost \,of \,mone{y}_{i}\right)}^{lifetime}-1)},$$

$$Cost\, of \,mone{y}_{i}\left(inflation \,adjusted\, cost\, of \,money\right)=\frac{1+Cost \,of \,money}{1+General\, inflation}-1.$$

The calculations for economic feasibility were based on assumptions from the literature and the Wadham facility reference case. Table 6 shows the assumptions for the economic analysis in this study. For the parameters related to the power plant operation, publicly available information on the characteristics of the Wadham facility were used^18,42^, and the capital expense (CAPEX) and the operating expense (OPEX) values were imported from a United States Energy Information Administration (U.S. EIA) report for a biomass power plant with a capacity of 50 MW in the United States^43^. The U.S. EIA report provides CAPEX for both Northern California and Southern California regions, and their average was used in this study. The report provides variable OPEX and fixed OPEX, and fuel cost was excluded from the variable OPEX. In this study, the fuel cost including the transportation cost may be assumed at or near zero depending on the location of a rice miller and a power plant. Rice hulls, the fuel feedstock in this study, are collected at milling sites regardless of their use or value, and thus, the transportation cost may be reduced by geographic location of a power plant. In addition, while market dynamics can fluctuate, rice hulls are not currently considered a high-value commodity and are used only in very low-value uses such as poultry bedding, which make rice hulls available at or near zero cost. The range of plausible costs were analyzed and discussed. As a cash flow method, discounted cash flow rate-of-return was used, and a straight line 30-year depreciation was assumed. For the cost of money, the weighted average cost of capital (WACC) for merchant-owned biomass facilities (7.21%) was used^44^. By using the WACC for biomass facilities as the cost of money for this study, the economic feasibility of this study compared to other biomass facilities can be determined.

**Table 6:** Assumptions for economic analysis.

	Unit	
Power plant operation		
Plant capacity	kW	29,100
Capacity factor	%	76.5
Net station efficiency	%	22.5
Expenses		
CAPEX	$/kW (2019)	4104
Variable OPEX (excluding fuel)	$/MWh (2019)	1.9
Fixed OPEX	$/kW-year (2019)	125.2
Other financial assumptions		
Federal tax rate	%	21
State tax rate (CA)	%	8.84
General inflation	%	2
Economic lifespan	years	30
Internal rate of return	%	7.21

### Experimental testing methods

To understand the feasibility of using RHA to make cement-based materials, experimental testing was performed on concrete specimens to measure key engineering design parameters, namely 28-day compressive and flexural strengths. RHA was obtained from the Wadham bioenergy facility in Williams, California (CA) owned by the Enpower Corporation. Lehigh Southwest Cement Company in Stockton, CA was the source of PC (ASTM type II/V). Both fine aggregates (99.95% passing a No.4 sieve, 4.75 mm) and coarse aggregates (with a 100% passing a 1″ sieve, 25 mm) were locally sourced from Esparto, CA.

Two concrete mixtures were made: a control mixture containing no RHA and a mixture with RHA replacing 15% of the PC by mass (Table 7). No chemical admixtures were used. For compressive strength and chloride ingress tests, 100 mm × 200 mm cylinders were prepared. Prismatic beams of 100 mm × 100 mm × 300 mm were made for flexural strength testing. All specimens were demolded one day after casting and then cured in a curing chamber at 25 °C and $\ge$ 80% of relative humidity until testing.

**Table 7:** Concrete mixture proportions.

	Portland cement (%)	Rice-hull ash (%)	Mixture proportions (kg/m^3^)				
			Portland cement	Rice-hull ash	Fine aggregate (< 4.75 mm)	Coarse aggregate (< 25 mm)	Water
100% PC	100	0	411	0	516	1141	193
15% RHA	85	15	349	61.6	493	1141	193

Compressive strength and flexural strength were determined after 28 days of curing. Compression tests were conducted on a SoilTest CT-950 load frame following ASTM C39^45^ where cylinder specimens were capped on either end with a neoprene-padded aluminum cap. Five specimens were tested for each mixture. The compressive strength of the concrete mixtures was determined using the maximum load before softening or failure occurred.

Flexural strength was determined by performing three-point bending tests, at 28 days. Testing was performed on an MTS Testline Component load frame managed by an MTS TestStarIIs controller following ASTM C293^46^. Three specimens were tested from each mixture and the flexural strength was determined using the maximum load prior to failure.

In addition to mechanical performance, a rapid chloride ingress test was performed to provide an initial indicator of the potential durability and longevity of the material. Chloride permeability reflects the ability for chloride ions to permeate into concrete and is a critical durability property for concrete used in certain regions. Chloride ingress in steel-reinforced concrete is a large contributor to the corrosion of steel. This is of particular interest in California due to saltwater exposure in coastal regions and predominant use of reinforcing steel in structural concrete. In this test, the resistance of saturated concrete specimens, with and without RHA, to chloride diffusion were measured. Concrete cylinders were cured for 90 days and then cut into disks. Measurements were collected using a PROOVE-it control unit and testing cells with external cooling fins, following ASTM C1202^36^.

### Life cycle assessment methods

To assess the potential environmental benefits of RHA replacing conventional PC or common SCMs, this study applies LCA to each material, RHA, PC, and coal fly ash, with a functional unit of 1 kg. The life cycle inventory (LCI) dataset for PC was obtained from the Ecoinvent 3.5 database using the GaBi software tool. However, reference LCI data for coal fly ash, which RHA replaces, were not available, and only LCIs for cement blends with 5–15% pozzolana and fly ash and 15–40% pozzolana and fly ash were available from the database. Thus, the environmental impacts were compared between the RHA cement mixture from this study and the cement blends from the database, instead of between RHA and fly ash. Also, the environmental impacts of RHA and electricity were based on a reference LCI for electricity from solid biomass, and they were allocated to RHA and electricity based on their relative economic values. Table 8 reports all the reference LCIs used in this study. Environmental impact potentials were calculated using the life cycle impact assessment method CML 2001^47^. The impact categories, characterization factors and indicator units are shown in Table 9.

**Table 8:** LCI data source (geographic region: United States) obtained from the GaBi software.

Name	Unit output	Source
Cement production, Portland	1 kg	Ecoinvent 3.5
Cement production, pozzolana and fly ash 5–15%	1 kg	Ecoinvent 3.5
Cement production, pozzolana and fly ash 15–40%	1 kg	Ecoinvent 3.5
Electricity from biomass (solid) (West)	1 kWh	Ecoinvent 3.5

Unit output as shown in the database.

**Table 9:** Characterization factors and indicator units of the CML 2001 (August 2016)^48^*.*

Characterization factor	Indicator unit
Abiotic depletion (elements) (ADP elements)	kg antimony (Sb) eq
Abiotic depletion (fossil resources) (ADP fossil)	MJ
Acidification potential (AP)	kg SO_2_ eq
Eutrophication potential (EP)	kg phosphate eq
Freshwater aquatic ecotoxicity potential (FAETP)	kg 1,4-dichlorobenzene (DCB) eq
Global warming potential (GWP 100)	kg CO_2_ eq
Human toxicity potential (HTP)	kg DCB eq
Marine aquatic ecotoxicity potential (MAETP)	kg DCB eq
Ozone layer depletion potential (ODP)	kg trichlorofluoromethane (R-11 or CFC-11) eq
Photochemical ozone creation potential (POCP)	kg ethene eq
Terrestrial ecotoxicity potential (TETP)	kg DCB eq

## Data Availability

Raw data supporting the conclusions of this article will be made available by the corresponding author upon request.

## References

[1] Turner LK, Collins FG (2013). Carbon dioxide equivalent (CO2-e) emissions: A comparison between geopolymer and OPC cement concrete. Constr. Build. Mater..

[2] Teh SH, Wiedmann T, Castel A, de Burgh J (2017). Hybrid life cycle assessment of greenhouse gas emissions from cement, concrete and geopolymer concrete in Australia. J. Clean. Prod..

[3] US Environmental Protection Agency (2022). Inventory of US Greenhouse Gas Emissions and Sinks: 1990–2020.

[4] United States Green Building Council. *LEED*. https://new.usgbc.org/leed (2021).

[5] Institute for Sustainable Infrastructure. *ENVISION* (2021).

[6] Rakhimova NR, Rakhimov RZ (2019). Toward clean cement technologies: A review on alkali-activated fly-ash cements incorporated with supplementary materials. J. Non Cryst. Solids.

[7] Huntzinger DN, Eatmon TD (2009). A life-cycle assessment of Portland cement manufacturing: Comparing the traditional process with alternative technologies. J. Clean. Prod..

[8] Giergiczny Z (2019). Fly ash and slag. Cem. Concr. Res..

[9] Erdoğdu K, Türker P (1998). Effects of fly ash particle size on strength of portland cement fly ash mortars. Cem. Concr. Res..

[10] Miller SA, John VM, Pacca SA, Horvath A (2018). Carbon dioxide reduction potential in the global cement industry by 2050. Cem. Concr. Res..

[11] California. *AB-2446 Embodied Carbon Emissions: Construction Materials* (2022).

[12] California. *SB-1020 Clean Energy, Jobs, and Affordability Act of 2022* (2022).

[13] California. *SB-596 Greenhouse Gases: Cement Sector: Net-Zero Emissions Strategy* (2021).

[14] California Air Resources Board. *California Greenhouse Gas Emission Inventory—2022 Edition* (2022).

[15] Caltrans. *Fly Ash: Current and Future Supply. A Joint Effort Between Concrete Task Group of the Caltrans Rock Products Committee and Industry*. http://www.dot.ca.gov/hq/esc/Translab/OSM/rpc_concrete_task_group/documents/Fly_Ash_Current_and_Future_Supply_Report_2016.pdf (2016).

[16] International Energy Agency (2009). Energy Technology Transitions for Industry—Strategies for the Next Industrial Revolution.

[17] Department of Finance State of California. *Projections: Population Projections (Baseline 2016)* (2017).

[18] California Energy Commission. California Biomass and Waste-To-Energy Statistics and Data. *California Energy Commission* https://www.energy.ca.gov/almanac/renewables_data/biomass/index_cms.php (2019).

[19] Kumar N (2018). Use of biomass ash for development of engineered cementitious binders. ACS Sustain. Chem. Eng..

[20] Munawar MA (2021). Challenges and opportunities in biomass ash management and its utilization in novel applications. Renew. Sustain. Energy Rev..

[21] Thomas BS (2021). Biomass ashes from agricultural wastes as supplementary cementitious materials or aggregate replacement in cement/geopolymer concrete: A comprehensive review. J. Build. Eng..

[22] Tosti L, van Zomeren A, Pels JR, Dijkstra JJ, Comans RNJ (2019). Assessment of biomass ash applications in soil and cement mortars. Chemosphere.

[23] Jittin V, Bahurudeen A, Ajinkya SD (2020). Utilisation of rice husk ash for cleaner production of different construction products. J. Clean. Prod..

[24] Siddika A, Mamun MA, Alyousef R, Mohammadhosseini H (2021). State-of-the-art-review on rice husk ash: A supplementary cementitious material in concrete. J. King Saud Univ. Eng. Sci..

[25] Cunningham PR, Wang L, Thy P, Jenkins BM, Miller SA (2021). Effects of leaching method and ashing temperature of rice residues for energy production and construction materials. ACS Sustain. Chem. Eng..

[26] Hu L, He Z, Zhang S (2020). Sustainable use of rice husk ash in cement-based materials: Environmental evaluation and performance improvement. J. Clean. Prod..

[27] Ozturk E, Ince C, Derogar S, Ball R (2022). Factors affecting the CO2 emissions, cost efficiency and eco-strength efficiency of concrete containing rice husk ash: A database study. Constr. Build. Mater..

[28] Fernando S (2021). Life cycle assessment and cost analysis of fly ash–rice husk ash blended alkali-activated concrete. J. Environ. Manag..

[29] United States Energy Information Administration. *State Electricity Profiles*. https://www.eia.gov/electricity/state/ (2018).

[30] United States Department of Agriculture. *USDA National Weekly Rice Summary*. https://mymarketnews.ams.usda.gov/viewReport/1655.

[31] BORAL. *Fly Ash Slides for Investors*. https://www.boral.com/sites/corporate/files/media/field_document/180528-Flyash-slides-for-investors-as-at-29May2018.pdf (2018).

[32] Oner A, Akyuz S, Yildiz R (2005). An experimental study on strength development of concrete containing fly ash and optimum usage of fly ash in concrete. Cem. Concr. Res..

[33] Mehta PK, Monteiro PJM (2006). Concrete: Microstructure, Properties, and Materials.

[34] United Nations. *Factsheet. The Ocean Conference* (2017).

[35] United Nations Department of Economic and Social Affairs Population Division. *The World’s Cities in 2018—Data Booklet* (2018).

[36] ASTM C1202 (2012). Standard Test Method for Electrical Indication of Concrete’s Ability to Resist Chloride Ion Penetration.

[37] Ganesan K, Rajagopal K, Thangavel K (2008). Rice husk ash blended cement: Assessment of optimal level of replacement for strength and permeability properties of concrete. Constr. Build. Mater..

[38] Ardente F, Cellura M (2012). Economic allocation in life cycle assessment. J. Ind. Ecol..

[39] Agrifood Consulting International. *Rice Value Chain Study: Cambodia*. http://www.fao.org/tempref/AG/Reserved/PPLPF/Docs/Reports&Papers/REP_MT_EA_AC_ValueChainRiceCambodia_ACI.pdf (2002).

[40] United States Department of Agriculture. *Rice Yearbook*. http://usda.mannlib.cornell.edu/MannUsda/viewDocumentInfo.do?documentID=1229 (2018).

[41] Enpower Corp. & Wadham Energy LP. *Enpower Corp.* http://www.enpowercorp.com/facilities/wadham-facility/ (2012).

[42] California Energy Commission. *California Power Plants*. https://www.energy.ca.gov/data-reports/energy-almanac/california-power-plants.

[43] U.S. Energy Information Administration. *Annual Energy Outlook 2020 with Projections to 2050*. https://www.eia.gov/outlooks/aeo/pdf/AEO2020FullReport.pdf (2020).

[44] Rhyne I, Klein J (2014). Estimated Cost of New Renewable and Fossil Generation in California.

[45] ASTM International (2017). Standard Test Method for Compressive Strength of Cylindrical Concrete Specimens.

[46] ASTM International (2002). Standard Test Method for Flexural Strength of Concrete (Using Simple Beam with Third-Point Loading).

[47] Guinee JB (2001). Life Cycle Assessment: An Operational Guide to the ISO Standards.

[48] Guinée JB (2002). Handbook on Life Cycle Assessment. Operational Guide to the ISO Standards. I: LCA in Perspective. IIa: Guide IIb: Operational Annex. III: Scientific Background.

